# Thermoresponsive Catechol Based-Polyelectrolyte Complex Coatings for Controlled Release of Bortezomib

**DOI:** 10.3390/ijms20236081

**Published:** 2019-12-02

**Authors:** Berthold Reis, David Vehlow, Tarik Rust, Dirk Kuckling, Martin Müller

**Affiliations:** 1 Leibniz-Institut für Polymerforschung Dresden e.V., Department Polyelectrolytes and Dispersions, Hohe Straße 6, 01069 Dresden, Germany; reis@ipfdd.de (B.R.); vehlow@ipfdd.de (D.V.); 2 Technische Universität Dresden, Department of Chemistry and Food Chemistry, 01062 Dresden, Germany; 3 Universität Paderborn, Department of Chemistry, Warburger Str. 100, 33106 Paderborn, Germany; trust@mail.uni-paderborn.de (T.R.); dirk.kuckling@uni-paderborn.de (D.K.)

**Keywords:** catechol chemistry, poly(caffeic acid), polyelectrolyte complex coatings, thermoresponsive coatings, controlled release, bortezomib, multiple myeloma

## Abstract

To overcome the high relapse rate of multiple myeloma (MM), a drug delivery coating for functionalization of bone substitution materials (BSM) is reported based on adhesive, catechol-containing and stimuli-responsive polyelectrolyte complexes (PECs). This system is designed to deliver the MM drug bortezomib (BZM) directly to the anatomical site of action. To establish a gradual BZM release, the naturally occurring caffeic acid (CA) is coupled oxidatively to form poly(caffeic acid) (PCA), which is used as a polyanion for complexation. The catechol functionalities within the PCA are particularly suitable to form esters with the boronic acid group of the BZM, which are then cleaved in the body fluid to administer the drug. To achieve a more thorough control of the release, the thermoresponsive poly(*N*-isoproplyacrylamide-*co*-dimethylaminoethylmethacrylate) (P(NIPAM-*co*-DMAEMA)) was used as a polycation. Using turbidity measurements, it was proven that the lower critical solution temperature (LCST) character of this polymer was transferred to the PECs. Further special temperature dependent attenuated total reflection infrared spectroscopy (ATR-FTIR) showed that coatings formed by PEC immobilization exhibit a similar thermoresponsive performance. By loading the coatings with BZM and studying the release in a model system, via UV/Vis it was observed, that both aims, the retardation and the stimuli control of the release, were achieved.

## 1. Introduction

Multiple myeloma (MM) is still one of the most deadly malign bone diseases, esteemed to cause the death of approximately 13,000 people in the USA in 2019 [1]. The relatively low five-year survival rate of 52.2% is due to the high relapse probability caused by the survival of the cancer cells in nearly impenetrable microlesions in the bone substance [1,2,3]. Modern chemotherapies try to tackle this problem through administration of high doses. On the one hand, this and the development of novel drugs have led to a prolongation of the average life expectancy in the last 15 years [3]. On the other hand, the high doses cause severe side effects, often weakening the patients gravely and thereby aiding the susceptibility towards relapses [3,4]. Due to these disadvantages and the persistently high death rate with the patients, MM is still considered to be an incurable disease [3,4].

Furthermore, nearly half of the MM patients suffer a pathologic fracture at some point during the course of the disease [5]. This derives from the cancer cells, which cause a displacement of the natural equilibrium between bone growth and bone resorption, leading to reduced bone stability. Hence, even modest tensions can result in serious fractures [6]. Because of the reduced self-healing capacity of the diseased bone, it is often necessary to restore the stability artificially via implantation of a bone substitution material [7].

A promising approach to overcome the barriers in MM therapy is the functionalization of bone substitution materials (BSM) with coatings that deliver potent drugs directly to the anatomical site of action [8,9,10,11]. The two major advantages that derive from such local drug delivery systems are esteemed to be the reduction of the required total doses and more efficient attacking of hidden cancer cells in peripheral microlesions [9]. The aim of this approach is to tackle both of the capital deficits of the modern applied therapies. 

To create a suitable coating for this biomedical application, various conditions have to be fulfilled. Firstly the coating has to exhibit a strong adhesiveness towards the bone substitution material under wet conditions [8,11,12,13]. Otherwise, instability of the layer can occur and the detached layer material and drug could be transported to different parts of the body, causing damage. Further, the key to a successful coating based therapy is a controlled release of the incorporated drug. To prevent harmful burst release, the coating should retain the drug [12]. Moreover it is essential, that the effective drug concentration at the site of action is held upright for the time the drug requires to establish its beneficial effect [12]. Both, the effective drug concentration and the required time of action are drug specific and depend on the respective pharmacokinetics and pharmacodynamics [13]. The corresponding coating has to be adjusted in its properties to match the characteristics of the specific drug that is to be released. Additionally it is advantageous, if the coatings are based on biocompatible materials, which includes avoiding toxic reactants and solvents during synthesis. 

Since the scale of requirements towards the applied coatings is very broad and can differ significantly with the respective drug, only highly alterable and adjustable systems qualify. Suitable systems are provided e.g., by polyelectrolyte complex nanoparticles (PEC NPs) [8,11,14,15]. In this approach, polyanions and polycations are complexed to nanoparticles in solution, which are subsequently immobilized to form the intended coatings. The resulting films tend to show nanostructured texturing with large surface areas and good wet adhesiveness towards various substrates (metal, plastics, ceramics etc.). By varying the mixing ratio of the polyelectrolytes, the net charge of the resulting complexes can be controlled. Anionic and cationic PEC NPs may exhibit strongly differing properties regarding binding and adhesion of drugs. Nearly neutral complexes are generally more suitable for immobilization due to their lower solubility in the rinse-and-release media (generally water) [15]. 

Another advantage is that a large number of different polyelectrolytes are available, through which various properties and functionalities can be inserted into the particles. PECs can therefore be understood as a kind of “toolbox” for creating nanoparticles with specific properties. Usually these properties are only marginally displaced during the immobilization process. Therefore, specific coatings matching individual drug-related requirements can be synthesized. 

One of the drugs that has significantly improved the therapy of MM is bortezomib (BZM) [4,16]. This boronic acid derivate functions as a proteasome inhibitor and exhibits great selectivity towards the MM cancer cells. On the downside, the administered high doses may lead to severe side effects, such as peripheral neuropathy and thrombocytopenia. By administration of BZM via a surface coating of BSM, these side effects are expected to be significantly reduced. The key to a successful therapy by this means is a controlled release of BZM from the coatings. To gain control over the release kinetics, the two previously mentioned conditions have to be fulfilled: Firstly, the burst release has to be prevented by incorporation of a drug retaining functionality and secondly, a stimuli-responsiveness has to be employed to implement an external control of the release properties to prevent a concentration decrease. 

In the present work, an acknowledged mechanism is tested to serve as a possibility for the retention of BZM. It is known that boronic acid derivates form boronic acid esters with diol functionalities under the separation of two water molecules. Springsteen et al. proved that these esters are even formed in aqueous solution and that aromatic diol functionalities tend to exhibit significantly larger equilibrium constants [17]. Based on this data caffeic acid (CA) was chosen to serve as the monomer for the used polyanion. CA includes a catecholic functionality as well as a carboxylate group, which is necessary for complexation. The diol functionality should serve as reactant for the formation of the boronic acid esters, which will be hydrolytically cleaved after transplantation into the body and therefore gradually release the BZM. Further CA is known as an ingredient of different food types and can therefore be considered a renewable feedstock [18]. Moreover it forms dimeric and trimeric products via autoxidation [18]. This reaction takes place in aqueous media, demonstrating that toxic organic solvents are not needed. Additionally, under harsher oxidative conditions, formation of higher oligomers has been observed, but until now has not been examined more detailed [19]. Often even short oligomeric products suffice to form PEC NPs that are stable against dissociation. The last major advantage of CA is that it can be classified as a catecholic compound, which have been reported to show strong adhesive properties [20]. Due to the high hydrophilicity of the charged carboxylic group CA and its oxidation products do not show adhesive properties when applied by itself. Complexation of the carboxylic group leads to a significant reduction of the hydrophilic character promoting the adhesive properties correlated with the catecholic functionality. 

**Figure 1:**
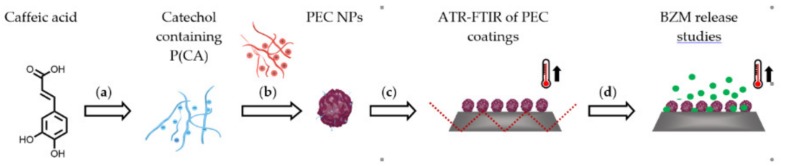
General procedure of first (**a**) oxidative coupling of CA; (**b**) complexation of PCA with the thermoresponsive P(NIPAM-*co*-DMAEMA); (**c**) Subsequent PEC immobilization to form coatings; (**d**) loading of the coatings with BZM for release studies.

The necessary complexation was exploited to establish an external control of the release. Therefore, P(NIPAM-*co*-DMAEMA) (22 mole% DMAEMA) was used as the polycation, which is a thermoresponsive copolymer that exhibits a lower critical solution temperature (LCST) at 55 °C [21]. The coatings from the PECs were analyzed regarding their thermoresponsive properties using a self-manufactured heatable ATR-FTIR measurement cell. Afterwards, a study of the BZM release kinetics was conducted. In total, the main aim of the study was to prove that PEC NPs coatings based on poly(caffeic acid) (PCA) and P(NIPAM-*co*-DMAEMA) are suitable to control the release of BZM. The complete procedure is schemed in Figure 1.

## 2. Results

In the following synthesis and molecular structure of PCA, preparation and properties of P(NIPAM-co-DMAEMA)/PCA complexes and finally release of the drug BZM from P(NIPAM-co-DMAEMA)/PCA complex coatings are described.

### 2.1. Synthesis of Poly(Caffeic Acid) (PCA)

When a caffeic acid (CA) solution was mixed with a NaIO_4_ solution, the resulting mixture changed its color from clear yellowish to black opaque within 10 s. This indicates an expansion of the π-system via coupling of either the aromatic and/or the conjugated R-C=C-R’ vinyl moieties. The structure of the yielded product was analyzed by various methods including dynamic light scattering (DLS), infrared spectroscopy (FTIR), electrospray ionization mass spectroscopy (ESI-MS), nuclear magnetic resonance (NMR) and colloid titration (PCD), aiming at determination of the molecular weight and closer insights into the coupling mechanism. 

Firstly, concerning the molecular weight of PCA, it was observed that during dialysis, using tubes with cut-offs up to 50 kDa, barely any product was eluted. This pointed to a considerable polymerization degree. However, since the related catechol compound poly(dopamine) is known to form aggregates in aqueous solutions, this is not necessarily an indication for high molar mass polymers [22]. No further insights were obtained by DLS, which revealed no distinct sizes around 2–1000 nm (due to low scattering contrast/intensity).

Secondly, PCD of the PCA product solutions (using low molecular probe polycation) revealed a charge factor of 1.0 ± 0.05, meaning that the quantity of charged groups of PCA is equivalent to the corresponding amount of CA monomers. Note that analogous colloid titration of CA monomer solutions did not reveal any related charge concentration value, since a phase-separated complex did not form, which is required for sensing principle. This observation is a further hint for the formation of a polymer with polymer-bound charges.

Thirdly, the FTIR spectrum (Figure S1) of the monomer (pH 9.0; under oxygen exclusion) shows four basic functionalities: the vinyl group (δCH 977 cm^−1^; νC=C 1635 cm^−1^), the aromatic ring structure (νC=C 1433 cm^−1^, 1495 cm^−1^, 1588 cm^−1^), the carboxylate group (νsCOO 1397 cm^−1^; νasCOO 1549 cm^−1^), which are obviously the origin of the polymer bound charges rationalized by PCD, and the phenolic functionality (νasCCO 1258 cm^−1^). All four functionalities are generally preserved during the course of the reaction, whereby especially the carboxylate signals remain widely unchanged. Generally, an increase of the bandwidth for the IR bands of the polymer was obtained, indicating restricted motions compared to the monomer. A strong decrease in intensity combined with a distinct increase of the width is especially observed at δCH 977 cm^−1^, suggesting a coupling via the vinyl functionality. The same observation, but less pronounced, can be made for the phenolic and aromatic functionalities. Here, the decrease of their intensities can be explained by the formation of quinones, whose corresponding signal appears at νC=O 1669 cm^−1^ as a shoulder [23]. Further the spectrum of the product does not show any ester signals. This was verified through PCD measurements, which rendered the charge factor of 1.0 ± 0.05 (see above) for the PCA, excluding coupling of the carboxyl and aromatic hydroxyl groups.

**Figure 2:**
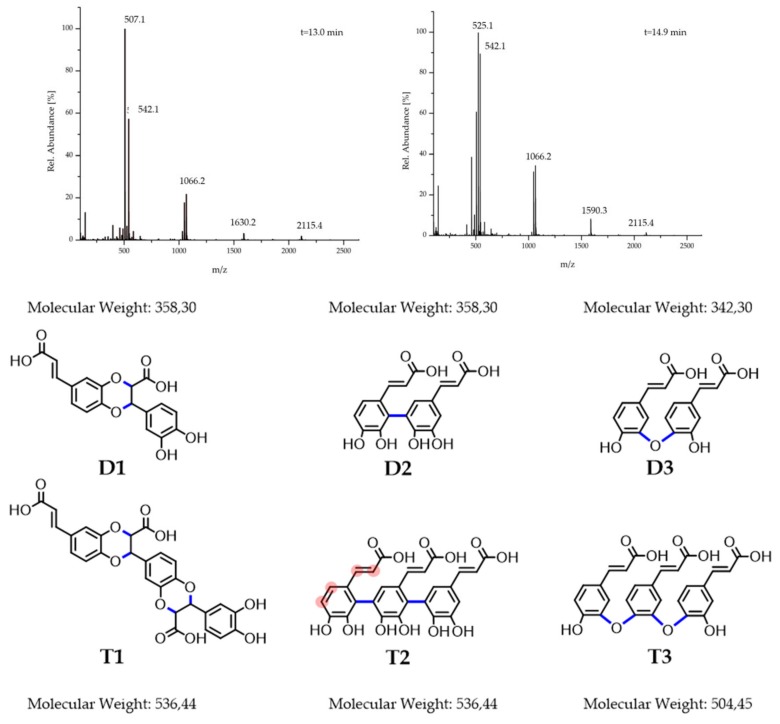
(Upper half) ESI mass spectra of the preferentially formed products. (Lower half) Proposed coupling types (formed bonds blue) consistent with the ^1^H-NMR and FTIR data shown using exemplary dimers (D) trimers (T): Coupling of the vinyl with the diol functionality (D1 + T1). Coupling via the π-system (D2 + T2 also possible at the red marked positions exemplary first monomeric unit T2). Coupling via a condensation mechanism (D3 + T3).

Fourthly, for further molecular structure determination, ^1^H-NMR spectra were recorded (Figure S2). The variety of broad and narrow peaks obtained point to a heterogeneous mixture of products with varying coupling types along chains of different lengths. The broad peaks of higher order between 6.70 and 8.00 ppm indicate the formation of polymeric structures and support the hypothesis concerning coupling of the π-system (Figure 2 D2 + T2). The multitude of different coupling positions along the π-system (Figure 2 T2, exemplary encircled red left monomeric unit) could explain the high number of different subproducts. The peaks in the range of 3.00–4.50 ppm indicate heteroatom substituted aliphatic compounds. As Arakawa et al. reported, the corresponding structures (Figure 2 D1 + T1) could be formed by an addition-reaction of the diol functionality to the vinyl group [19]. The loss of the phenolic and vinyl groups are consistent with the decrease of their corresponding IR signals. Since the vinyl group is still clearly visible at 6.45 ppm, this specific coupling mechanism can only partially be held accountable for product formation. By comparing the integral intensities of the 3.00–4.50 ppm signals, deriving from two protons, with the integral of the signal at 6.45 ppm, deriving from one vinylic proton, it becomes clear that about half of the products are formed via the addition mechanism. This is further supported by the remaining phenolic and vinylic IR signals, proving that part of the, for BZM binding essential, diol functionalities outlast the polymerization process. 

Finally, reverse phase liquid chromatography ESI-MS was applied for further clarification. The chromatogram confirms the formation of a broad spectrum of products (Figure S3) analogously to the NMR findings. Some single peaks protrude from the underlying wide spectrum, which implements the favored formation of particular products. The ESI mass spectra of the five major formed compounds (elution times 13.0; 14.9; 18.4; 19.2 and 22.0 min) were analyzed. The elution peaks at 18.4 min and 19.2 min are caused by dimeric structures (Figure S4a,b). The peak at 359 *m/z* is consistent with the coupling of the double bond and the diol functionality or a π–π coupling, since both possibilities form dimers of 358 g/mol (Figure 2 D1 + D2) The peak at 341 *m/z* on the other hand points towards a condensation mechanism with a separation of a water molecule (Figure 2 D3). This is supported by the peak difference of 18 *m/z* (360 *m/z* – 341 *m/z* – H^+^ = 18 *m*/*z*), which matches the molecular weight of water. The elution peak at 22.0 min shows several trimeric units (Figure S4b). This coincides with the observations from Arakawa et al. who found that trimeric products are generated when CA is oxidized electrochemically at higher voltages [19]. 

Of even higher significance are the mass spectra of the elution peaks of 13.0 min and 14.9 min in Figure 2. Neither of the two spectra shows peaks or peak-differences corresponding to a monomeric unit of 180 g/mol. Instead, all of the lowest peaks are positioned at around 540 *m*/*z*, again indicating the formation of trimeric products. The peaks at higher *m/z* ratios are proximate to the many-folds of the trimeric values. Many peaks of the spectra at an elution time of 14.9 min are around 525 *m/z* apart (e.g., 2115.4 − 1590.3 = 525.1). Since no change of the *m/z* value is observed, this may point towards an aggregation of 525.1 *m/z* trimers. The peak differences that do not correspond to any *m/z* peak indicate a chemical reaction and thereby can be attributed to the formation of longer oligomers (t = 13.0 min). For these, a cascade of two different mechanisms is proposed: first, a trimerization of the CA occurs, followed by a subsequent coupling of these trimers to higher oligomers. Even though different coupling types are proposed, it is not distinguishable if all types are responsible for trimerization and the subsequent coupling to longer oligomers. Strikingly, often the spans between neighbored peaks is 18 *m/z* or 17 *m/z* (e.g., 525–507 m/z; 542–525 m/z) supporting the condensation mechanism under cleavage of water. Since no ester IR signals or reduction in the charge factor was observed, only ether bonds with IR bands overlapping with the phenolic bands serve as a suitable explanation. Furthermore, if two oligomers with a molar mass of 1066.2 g/mol are coupled via this condensation-mechanism, an oligomer with a corresponding molar mass to the peak at 2115.4 *m/z* is yielded (2 × 1066.2 *m/z* – 18 *m/z* = 2115.4 *m/z*). This indicates that this mechanism may also be suitable to combine the trimers with each other. 

Due to the complexity of the product mixture, further investigations are necessary for profound structural proposals, and the trimeric structures in Figure 2 should rather be understood as suggestions. However, from the synthetic part given above, it can be concluded that upon oxidation of the CA oligomeric structures were formed under partial preservation of the aromatic diol functionalities, which are important for chemically binding boronic acid-containing drugs, like BZM. 

### 2.2. Preparation and Characterization of P(NIPAM-co-DMAEMA)/PCA Complexes

From our former work it is known, that polyelectrolyte complex nanoparticles (PEC NP) prepared by molar mixing ratios close to 1.0 (see Experimental) show high wet-adhesiveness, which arises from lack of both polymer-bound excess charge and hydrophilicity [15]. By gradually increasing or decreasing the ratio by 0.1 increments, cationic and anionic PEC NPs were determined, which form a colloidal stable dispersion for at least 5 h. Regardless, the quantity of excess PCA in the anionic PEC system (i.e., *n*−/*n*+ > 0, see Experimental) was stable. This is probably due to the high difference in the molar mass of the polyelectrolytes, so that the anionic groups of the significantly shorter PCA oligomers are fully compensated by the cationic groups of the larger P(NIPAM-co-DMAEMA) and thus do not protrude and exhibit an excess charge in the shell region of the PEC NP [24]. Hence, the lack of such (anionic) excess charge disfavors repulsive forces between the PEC NPs resulting in flocculation even at mixing ratios significantly higher than *n*−/*n*+ = 1.0 (tested up to 3.0). However, when vice versa the cationic P(NIPAM-co-DMAEMA) is the excess component, longer polycationic sections are protruding at the shell region of PEC NP, which cause interparticle electrostatic repulsion and thus result in stable PEC solutions even at mixing ratios *n*−/*n*+ = 0.9.

**Figure 3:**
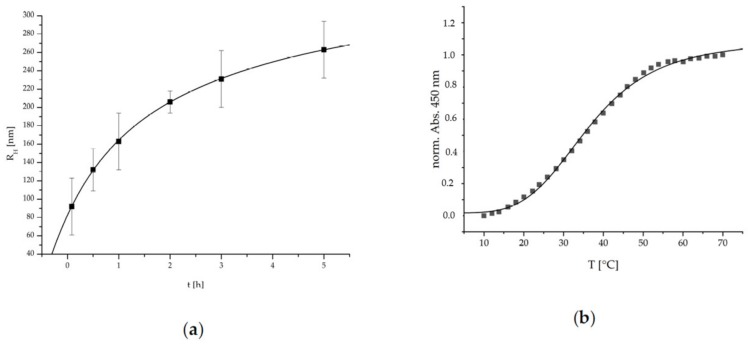
(**a**) Time dependent hydrodynamic radii (R_H_, via DLS measurement) and (**b**) turbidity measurement of P(NIPAM-co-DMAEMA)/PCA.

The ripening and growth behavior of such cationic PEC NPs system was analyzed through time-dependent DLS measurements. In Figure 3, the determined hydrodynamic radii are plotted against the time.

The PEC NPs show a significant exponentially damped (i.e., R_H_ = R_H_^0^ (1 − exp[−k t])) increase of the hydrodynamic radii R_H_ over time. This is possibly due to the dynamic competition between long range electrostatic repulsion (see above) and short-range attraction forces between initially formed primary PEC NP like Van der Waals or hydrogen bonding preventing thermodynamic equilibrium and suggesting kinetic control known for other PEC NP systems [25]. Polyelectrolyte complexation kinetics has been addressed in more detail by Takahashi and Liu based on time-resolved ultra-small-angle X-ray scattering supporting aggregation processes of early formed primary complexes to species termed higher order agglomerates or condensed coacervate droplets [26,27]. Practically, to assure reproducible measurements the solutions were always used after 1 h ripening for subsequent analysis or coating generation.

Recently, the thermoresponsive character of PEC systems based on cationic P(NIPAM-co-DMAEMA) and anionic polysaccharides was described [20]. To evaluate the thermoresponsive properties of the herein used P(NIPAM-co-DMAEMA)/PCA PEC NP system, temperature dependent UV/Vis measurements were conducted. In Figure 3, the normalized absorption at 450 nm is plotted against the temperature. The error due to increasing turbidity caused by the particle ripening was limited to 5% by time-dependent UV/Vis measurement at RT. 

The P(NIPAM-co-DMAEMA)/PCA PEC NP show a typical S-shaped function, which can be represented by a sigmoidal fitting function given therein [20]. Via this fit a conversion temperature (T_c_) of 36.4 ± 0.4 °C and a cooperativity factor (P) of 4.2 ± 0.2 were determined. Concerning the intended application, this LCST behavior is well-suited to match the requirements since it is located in the physiological temperature range. The relatively low P-factor is an indicator for a wide transition span, the advantage being that the complete range of physiological temperatures exhibits a thermoresponsive transition. Additionally, this system is also suitable if medicinal means are utilized that enable the application of higher temperatures such as hyperthermia therapies, etc. 

#### 2.2.1. Wet Adhesive Properties

In the preceeding section, thermoresponsiveness was observed for the bulk P(NIPAM-co-DMAEMA) PEC system in dispersion. To check interfacial thermal properties, this PEC system was deposited onto germanium crystals, which are both suitable analytical substrates of choice for surface sensitive ATR-FTIR measurements (see Experimental Section, Immobilization of PEC NPs) as well as model substrates for a series of oxide and hydroxide layer terminated metal and semiconductor materials (titania, steel, magnesium etc.) used as BSM. 

**Figure 4:**
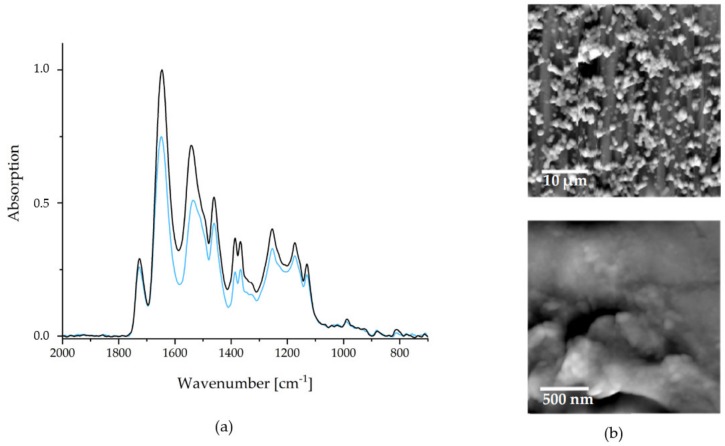
(**a**) FTIR spectra of the PEC NP coating P(NIPAM-co-DMAEMA)/PCA before (black) and after (blue) rinsing. (**b**) SFM images of the dry coating after rinsing.

As mentioned above, the wet adhesiveness of the PEC NPs towards a substrate is one of the most significant mandatory properties of biofunctional and biomedical organic coatings of BSM concerning future applications. As a simple test for wet-adhesiveness, FTIR spectroscopy was used, whereby the integrals of the IR signals before (A_BEFORE_) and after intense rinsing (A_AFTER_) in selected aqueous media are compared. Wet adhesiveness in that sense can be quantified calculating the percentage ratio R_ADHES_ = (A_AFTER_)/(A_BEFORE_) × 100%. Typical FTIR recorded before (black) and after (blue) are depicted in Figure 4.

Ultrapure water was applied as rinse medium, since it was empirically found to be the most erosive for PEC NP coatings. An average wet adhesiveness of the P(NIPAM-co-DMAEMA)/PCA PEC coating of R_ADHES_ = 79 ± 10% was determined. The deviation to 100% can be explained by a small amount of unbound excess polycation (see comments above). Consequently, coated implants and bone substitution materials are ready for use after one short rinsing step. 

Due to its impact on the release kinetics, the surface morphology is also evident for the quality of a drug delivery coating. In Figure 4, SFM images are depicted, with the larger section (microscale) on the upper and the smaller section (nanoscale) on the lower hand. 

As it can be seen on the microscale, the PCA system strongly tends to cluster formation. These clusters coagulate to an island-like structure, rendering a larger surface area than a smooth coating would. The advantage of a high surface area is, that more, easily accessible, interaction sites for drug binding are present. This also depends on the nanostructures of the films, which are depicted in the lower image in Figure 4. The NPs, which were observed in solution via DLS measurements, are recognizable. Furthermore, agglomeration and coalescence of these NPs can be observed. The average diameters of individually appearing NPs (range 60–180 nm) were significantly lower than the corresponding values in dispersion (Figure 4). This indicates a shrinking of the particles during the drying process and suggests a certain swelling of the PEC NP in the dispersed state.

#### 2.2.2. Thermoresponsive Properties

**Figure 5:**
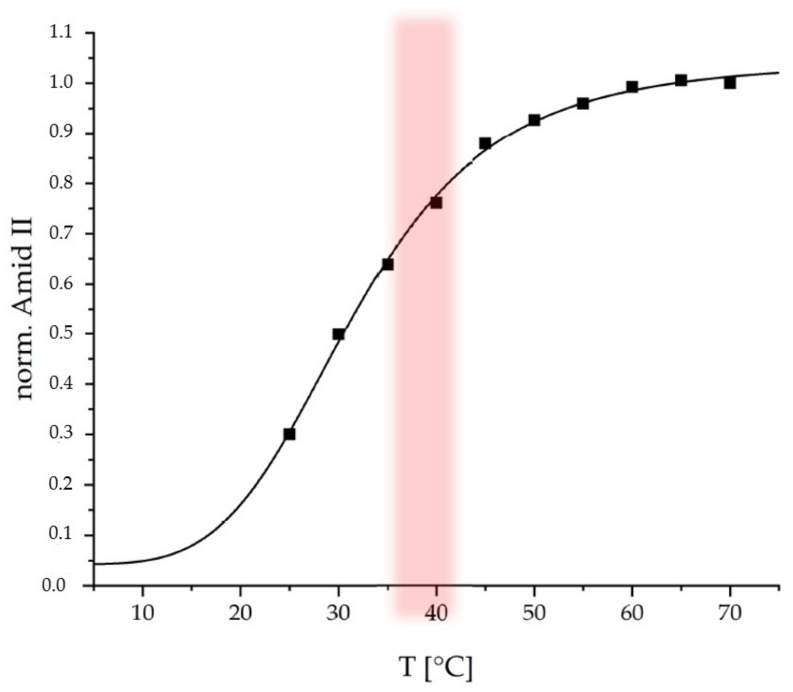
Temperature dependent ATR-FTIR measurements of P(NIPAM-co-DMAEMA)/PCA; physiological temperature range red.

Subsequently the coating was tested regarding its thermoresponsive properties at a physiological pH of 7.4. Temperature-dependent ATR-FTIR spectroscopy was used. In the Figure 5 the normalized integrals (with respect to end value) of the Amide II band from ATR-FTIR spectra (Figure S5) recorded at 25 °C to 70 °C are plotted versus temperature. 

The Amide II band is used, since it is not interfered by the δ(OH) band of water around 1640 cm^−1^, which is the case for the Amide I band. An increase of the Amide II (1560 cm^−1^) integral with increasing temperature is observed for the thermoresponsive PEC coating, which is similar to that observed for the thermoresponsive PEC NP dispersion (Figure 3b). On the one hand, increasing ATR-FTIR signals may originate from increasing thickness within a range up to the penetration depth (d_P_ < 500 nm dependent on refractive indices of contributing media and incident angle of ATR crystal) of the evanescent wave [28]. On the other hand, increasing ATR-FTIR signals may originate from increasing density or polymer segment concentration c_POL_. Since it is known that PNIPAM systems rather undergo a shrinking process upon temperature increase, the curve in Figure 5 rather monitors c_POL_. This curve was fitted by the same sigmoidal function used to fit the dispersion data in Figure 3b. For the PEC NP coating, a conversion temperature T_C_ = 31.7 ± 1.5 °C and a cooperativity factor of P = 4.4 ± 0.6 were determined. Both values for the thermoresponsive PEC coating are close to those for the PEC NP dispersion suggesting that the immobilization process does not have a significant effect on the response behavior. The slightly lower T_C_ for the coating compared to the dispersion might be caused by the lower water accessibility (hydratability) in case of the coating due to lower surface area and removal of hydrophilic excess polycation. 

### 2.3. Release of BZM

**Figure 6:**
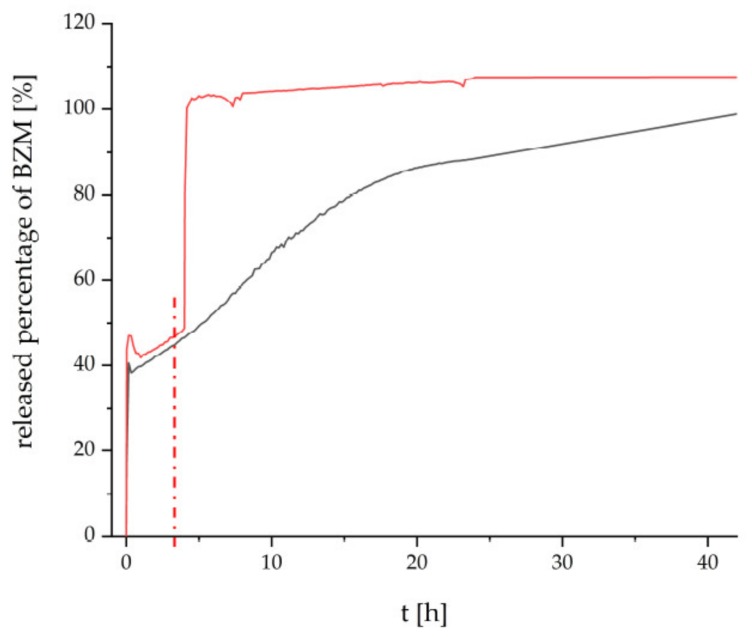
Release of BZM from the P(NIPAM-co-DMAEMA)/PCA coating in ultrapure water at RT (black) and under temperature increase from RT to 42 °C (red) after 4 h (dashed line).

Finally, the thermoresponsive PEC NP (P(NIPAM-co-DMAEMA)/PCA) coating were checked for their drug delivery properties. Bortezomib (BZM) a potent drug for multiple myeloma (MM) described in the introduction was used, which bears a boronic acid moiety. BZM was loaded after preparation of the PEC NP coating. This “postloading” concept (section “BZM loading and release”) features two major advantages. Firstly, it is fast and easy to apply, which makes it appealing for commercial use. Secondly, the drying step is supposed to withdraw water from the equilibrium between the boric acid and the boric acid ester and to promote the ester formation. The dry coating with the bond BZM was subsequently placed into the release media, which was constantly monitored via UV/Vis measurements. In Figure 6, the released percentage of BZM is plotted versus time.

The release at room temperature (RT) from the PCA based coating is shown in Figure 6 by the black curve. The beginning is marked by an initial burst release of about 40%, which is most probably caused by loosely bound BZM without chemical bonding to the coating. The following slight decrease might be caused by reabsorption due to swelling of the coating. Thereafter the remaining BZM is gradually released over a time period until 42 h. We assume that the high tendency to form esters between the aromatic diol functionalities and the BZM is mainly accountable for this retardation of the release. The heterogeneous surface morphology, which is destined to provide a distinct number of interaction sites, supposedly amplifies the retention mechanism. Additionally, ATR-FTIR spectra were recorded on the loading and releasing process at an equivalent P(NIPAM-co-DMAEMA)/PCA coating at Ge substrate, which are given in Figure S6 of the Supplementary. There it can be rationalized that BZM is uptaken at this thermoresponsive PEC coating and it could not be completely rinsed out after rinsing in pure water. However, this data cannot give further information on the location of loaded BZM (shell, core or between particles) within the PEC coating. 

The release beginning with RT and after switching to T = 42 °C is given by the red curve in Figure 6. Obviously, at RT, the PEC NP coating elutes BZM with the same kinetics for 4 h. However after switching to T = 42 °C (switching point is denoted as red dashed line) the BZM is released much faster compared to RT (black line) up to 42 h. Analogously to other PNIPAM-based systems (e.g., pure polymer, crosslinked microgels), the known coil-to-globule transition of the PNIPAM segments causes conformation changes not only within the cationic P(NIPAM-co-DMAEMA), but also within anionic PCA [29]. Consequently, either physically entrapped BZM may leave the thermoresponsive PEC NP phase on new routes or residual water molecules may access to BZM-diol ester sites, which were previously shielded. 

## 3. Discussion

The aim of the study was to principally prove that polymeric oxidation products of the natural occurring catecholic compound caffeic acid are suitable to be integrated into adhesive, thermoresponsive PEC coatings and there act as a restraining functionality for the MM drug BZM. Thereby, the approach followed a line of subordinate steps, which are provided in the following.

In the synthetic part of this report oxidative products were identified by various analytical techniques and a mechanism proposed. The formation of dimers, trimers and oligomers was observed. Three types of couplings are proposed to occur during reaction: (i) At first, conjugation of the π-system may be proposed due to coloring of the reaction mixture, broadening of FTIR and NMR signals and specific *m/z* values of the MS spectra. We assume this to be based on the recombination of mesomerically stabilized radicals, since similar proposals have already been reported [19]. (ii) Secondly, an addition mechanism of the diol functionality to the vinyl group was observed supported by NMR measurements. These products have already been reported and may form by a mechanism similar to a hetero-Diels–Alder reaction. Thirdly, a coupling via a condensation of two hydroxyl functionalities is suggested based on the ESI mass spectra, which exhibit several peak differences of 18 *m/z* indicating the cleavage of a water molecule. Of greater significance is that the oligomers are composed of different trimers. Two separate mechanisms are proposed, primary a trimerization occurs and subsequently, these trimers are coupled to longer oligomers. Further investigations are necessary to support and substantiate these preliminary propositions.

By complexation of PCA with thermoresponsive P(NIPAM-*co*-DMAEMA) colloidal stable PECs with a cationic excess (ratio 0.9) were obtained. DLS measurements showed that the particles undergo ripening, thereby growing in radius over time. This is assumed to be caused by secondary short range interaction forces between small primary PEC NP. Furthermore, thermoresponsive behavior of the PECs was observed via turbidity measurements, whereby a physiologically relevant conversion temperature T_c_ of 36.4 ± 0.4 °C was found

Subsequent deposition of dispersed PEC NP lead to coatings with a wet adhesiveness of around 80%. The analysis via SFM showed high structured surfaces on both the micro and the nanoscale. These can be considered advantageous due to their amount of possible drug binding sites. Additionally, temperature dependent ATR-FTIR measurements proved that the thermoresponsive character of the PEC NP dispersion is preserved for the PEC NP coating featuring only a slight downshift of the conversion temperature (T_c_ = 31.7 ± 1.5 °C) due to less hydratability.

BZM could be successfully loaded into P(NIPAM-*co*-DMAEMA)/PCA coatings and released in a delayed manner within 42 h. Finally, a temperature stimulus from RT to T = 42 °C resulted in an accelerated elution of BZM due to local conformation and accessibility changes.

In general, PCA-based systems are proven not only to be wet adhesive and stimuli-responsive, but also to release chemically bound drugs on demand, although temperature-based drug delivery systems are challenging for clinical applications, if the physiological temperature range is exceeded.

## 4. Materials and Methods 

### 4.1. Chemicals

**Figure 7:**
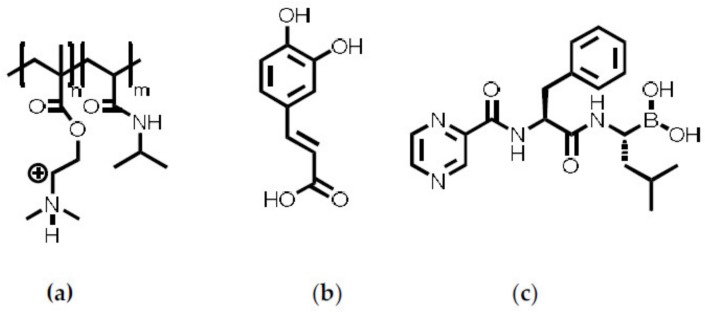
Structures of (**a**) P(NIPAM-*co*-DMAEMA), (**b**) CA and (**c**) BZM.

The random copolymer P(NIPAM-*co*-DMAEMA) (Poly(*N*-isopropylacrylamide-co-dimethylaminoethyl methacrylate), M_n_ = 7.200 g/mol, NIPAM 78 mole%, DMAEMA 22 mole%) was synthesized by RAFT polymerization [20] (Figure 7). The Bortezomib (BZM, M = 384.24, g/mol, >98%, Hycultec GmbH, Vilshofener Str. 35, 94501 Beutelsbach, Germany) was used as purchased. Further, the caffeic acid (CA, M = 180,16 g/mol, ≥ 98%), the 4-(2-hydroxyethyl)-1-piperazineethanesulfonic acid (HEPES, M = 238.31 g/mol, ≥ 99.5%) and the sodium metaperiodate (NaIO_4_, M = 213.89 g/mol, ≥ 99.5%) where obtained by Sigma Aldrich (Merck KGaA, 64293 Darmstadt, Germany) and used without further purification. For solving and dilution, ultrapure water (Milli-Q Advantage A10 (Merck KGaA, 64293 Darmstadt, Germany)) was used.

### 4.2. Oxidative Polymerization of Caffeic Acid

In a 250 mL round-bottom flask, equipped with a septum and a magnetic stirrer, ultrapure water (150 mL) was purged with nitrogen (N_2_) for 1 h. Subsequently the CA (135 mg; 0.75 mmol) was added and the pH was adjusted to 9.5 ± 0.2 via addition of NaOH (1 M) under N_2_ counterflow. The solution was purged for another 15 min before adding NaIO_4_ (8 mg; 0.15 mmol) solved in 1 mL ultrapure water. The reaction mixture was stirred for 3 d at room temperature (RT). Afterwards the solution was filled into dialysis tubes (12–14 kDa cutoff) and dialyzed against neutral, ultrapure water for 5 d (daily media exchange). The purified solution was lyophilized to afford the blackisch/brown product, which was characterized through ^1^H-NMR, ^13^C-NMR (Avance III 500 MHz, Bruker Biospin; in D_2_O), TRANS-FTIR (Tensor II Bruker-Optics GmbH, Rudolf-Plank-Straße 27, 76275 Ettlingen, Germany) dried on Ge-substrate and LC-ESI-MS (Agilent 1260 Infinity II coupled with 6230B Time of Flight (TOF) LC/MS, Agilent Technologies Deutschland GmbH, Hewlett-Packard-Straße 8, 76337 Waldbronn, Germany) measured in 1:1 H_2_O/ACN medium at 175.0 V).

### 4.3. Synthesis of the PEC NPs

The different polyelectrolytes were solved in ultrapure water to generate the required solutions (0.01 M). The charge factors where determined through PCD measurements (Mütek PCD-04, BTG Instruments GmbH, Arzberger Str. 10, 82211 Herrsching, Germany). The PEC NPs were synthesized with different mixing ratios, which is described using Equation (1). The charge factors F−/+ describe the charged proportion of the monomeric units, while *n*−/+ is the amount of substance (mol) of the polyanion and polycation, respectively.

(1) $$a\;=\;\frac{F^{-}\cdot \;n^{-}}{F^{+}\cdot \;n^{+}}$$

The solution of the excess component was presented and that of the minority one was added dropwise (at 600 rpm). This order in the course of the preparation was crucial to prevent flocculation. In case of the synthesis of cationic ratios, the pH of the presented P(NIPAM-*co*-DMAEMA) solution was adjusted to 5.5 ± 0.2 in advance to assure that all dimethylamino functionalities where charged. After merging the oppositely charged solutions, the resulting mixture was stirred for 15 min. Afterwards the pH was adjusted to 7.0 ± 0.1 by addition of HCl (0.1 M). The colloidal PEC NPs were analyzed by DLS (Jianke Portable Particle Sizer, Jianke Instruments Co. Ltd., Wuhu, P.R. China) and temperature-dependent UV/Vis measurements on a JASCO V-650 spectrometer (JASCO Deutschland GmbH, Robert-Bosch-Straße 14, 64319 Pfungstadt, Germany).

### 4.4. Immobilization of PEC NPs

100 µL of the colloidal PEC NP solutions were casted on a rectangular area of 1 × 1 cm on either a larger trapezoid germanium model substrate (area: 50 × 20 mm^2^, for analysis) or a smaller germanium model substrate (area: 20 × 10 mm^2^ for release studies) and dried on a heated metal block at 50 °C. The dry coating was rinsed in ultrapure water for 1 h during which the rinse-medium was exchanged two times after 10 min, respectively. To determine the adhesiveness the coatings were measured by TRANS-FTIR (see above) before and after the rinsing process. Further the coatings were analyzed by ATR-FTIR (SBSR device, Optispec, Rigistrasse 5, 8173 Neerach, Switzerland, attached to a FTIR spectrometer, Tensor II Bruker-Optics GmbH, Rudolf-Plank-Straße 27, D-76275 Ettlingen, Germany) dried on Ge-substrate and by SFM (Nanostation II, Bruker Nano GmbH, Am Studio 2d, 12489 Berlin, Germany) as it was described therein [15].

### 4.5. BZM Loading and Release 

A solution of BZM (0.5 mL, 0.25 mM) was casted on the rinsed and dried PEC films on the smaller Ge model substrates. The substrate was placed in a desiccator and the solution dried at RT by slow application of vacuum. The release was initiated by placing the substrates with the BZM-loaded films into a UV/Vis cuvette (10 mm, QS, Hellma Optik GmbH, Mühlenstraße 30, 07745 Jena, Germany) filled with 3 mL ultrapure water. The release was monitored by in situ measurement of the absorption at 270 nm using UV/Vis spectrometer (JASCO V-650, see above). To eliminate cuvette effects, a baseline and the absorption at 350 nm were subtracted from the spectra and the value at 270 nm, respectively. Furthermore, the temperature of the release media was controlled using a Peltier element. To determine the released amount of BZM a calibration curve was measured, using the same procedure as mentioned above. A linear dependency between concentration C and absorption A was observed. The corresponding Equation (2) (R^2^ = 0.9991) is shown below. 

(2) A=8.7332·c+0.0331

The maximum concentration (C_MAX_) at total release of BZM is equal to the ratio of the volume of the loading solution (V_LOAD_) to the volume of release media (V_RELEASE_) multiplied with the initial concentration (C_BZM_), what yields a value of 0.04167 mM (Equation (3)).

(3) $$c_{MAX}=\frac{V_{LOAD}}{V_{RELEASE}}\cdot \;c_{BZM}$$

Using Equation (2), the maximum absorption can be calculated (A_MAX_ = 0.397). The maximum absorption can be used to determine the percentage of released BZM-amount χ.

(4) $$\chi =\frac{A}{A_{MAX}}\cdot 100\;\%=\frac{A}{0.397}\cdot 100\;\%$$

The calculated released amount was then plotted against the time.

## 5. Conclusions

A drug-delivery coating based on adhesive, catechol-containing and stimuli-responsive polyelectrolyte complexes (PEC) is reported, which is designed to release the multiple myeloma (MM) drug bortezomib (BZM) at the anatomical site of action. BZM and catechol groups form intermediate ester groups aiming at the sustained cleavage reaction in contact to body fluid. As the catechol compound and the naturally occurring caffeic acid (CA) was coupled oxidatively to form poly(caffeic acid) (PCA), bearing still active catechol groups. The synthesized polyanion PCA was complexed by the thermoresponsive polycation poly(*N*-isoproplyacrylamide-co-dimethylaminoethylmethacrylate) (P(NIPAM-co-DMAEMA)) aiming at an on-demand release of BZM. Using turbidity measurements it was proven, that the lower critical solution temperature (LCST) character of this copolycation was transferred to the PEC system (PNIPAM-co-DMAEMA/PCA). Further special temperature-dependent attenuated total reflection Fourier transform infrared (ATR-FTIR) spectroscopy showed that coatings formed by PEC immobilization exhibit a similar thermoresponsive performance. By loading the coatings with BZM and studying the release in a model system via UV/Vis, it was observed that both aims were achieved: the retardation and the stimuli control of the release.

## Supplementary Materials

Supplementary Materials can be found at https://www.mdpi.com/1422-0067/20/23/6081/s1.

## Abbreviations

ATR-FTIR	attenuated total reflectance Fourier transform infrared spectroscopy
BSM	bone substitution material
BZM	bortezomib
CA	caffeic acid
DLS	dynamic light scattering
Ge	germanium
IR	infrared
LCST	lower critical solution temperature
MM	multiple myeloma
NMR	nuclear magnetic resonance
PCA	poly(caffeic acid)
PCD	particle charge detector
PEC NP	polyelectrolyte complex nanoparticle
PEL	polyelectrolyte
P(NIPAM-*co*-DMAEMA	poly(*N*-isopropylacrylamide-co-dimethylaminoethyl methacrylate)
RAFT	reversible addition–fragmentation chain-transfer
RT	room temperature
SFM	scanning force microscopy
UV/Vis	ultraviolet–visible spectroscopy
VPTT	volume phase transition temperature

## References

[1] Myeloma—Cancer Stat Facts. https://seer.cancer.gov/statfacts/html/mulmy.html.

[2] Saad F., Lipton A., Cook R., Chen Y.-M., Smith M., Coleman R. (2007). Pathologic fractures correlate with reduced survival in patients with malignant bone disease. Cancer.

[3] Girnius S., Munshi N.C. (2013). Challenges in multiple myeloma diagnosis and treatment. Leuk. Suppl..

[4] Paramore A., Frantz S. (2003). Bortezomib. Nat. Rev. Drug Discov..

[5] Melton L.J., Kyle R.A., Achenbach S.J., Oberg A.L., Rajkumar S.V. (2004). Fracture Risk with Multiple Myeloma: A Population-Based Study. J. Bone Miner. Res..

[6] Utzschneider S., Schmidt H., Weber P., Schmidt G.P., Jansson V., Dürr H.R. (2011). Surgical therapy of skeletal complications in multiple myeloma. Int. Orthop..

[7] Maurer F., Ambacher T., Volkmann R., Weller S. (1995). Pathologische frakturen: Diagnostische und therapeutische überlegungen bowie behandlungsergebnisse. Langenbecks Arch. Chir..

[8] Muller M., Vehlow D., Torger B., Urban B., Woltmann B., Hempel U. (2018). Adhesive Drug Delivery Systems Based on Polyelectrolyte Complex Nanoparticles (PEC NP) for Bone Healing. Curr. Pharm. Des..

[9] SFB Transregio 79—Werkstoffe für die Geweberegeneration im Systemisch Erkrankten Knochen. https://www.uni-giessen.de/fbz/fb11/forschung/schwerpunkte/sfb/trr79/index.

[10] Müller M. (2013). Method for Producing a Drug Delivery System on the Basis of Polyelectrolyte Complexes. U.S. Patent.

[11] Müller M., Urban B., Vehlow D., Möller M.L. (2017). Adjusting and switching the elution of bone therapeutics from thermoaddressable coatings of poly(N-isopropylacrylamide-co-acrylic acid)/ethylenediaminocellulose complex particles. Colloid Polym. Sci..

[12] Liechty W.B., Kryscio D.R., Slaughter B.V., Peppas N.A. (2010). Polymers for Drug Delivery Systems. Annu. Rev. Chem. Biomol. Eng..

[13] Holford N.H.G., Sheiner L.B. (1982). Kinetics of pharmacologic response. Pharmacol. Ther..

[14] Müller M., Kessler B. (2012). Release of pamidronate from poly(ethyleneimine)/cellulose sulphate complex nanoparticle films: An in situ ATR-FTIR study. J. Pharm. Biomed. Anal..

[15] Müller M., Urban B., Schwarz S. (2018). Biorelated Polyelectrolyte Coatings Studied by in Situ Attenuated Total Reflection–Fourier Transform Infrared Spectroscopy: Deposition Concepts, Wet Adhesiveness, and Biomedical Applications. Langmuir.

[16] Richardson P.G., Sonneveld P., Schuster M.W., Irwin D., Stadtmauer E.A., Facon T., Harousseau J.-L., Ben-Yehuda D., Lonial S., Goldschmidt H. (2005). Bortezomib or high-dose dexamethasone for relapsed multiple myeloma. N. Engl. J. Med..

[17] Springsteen G., Wang B. (2002). A detailed examination of boronic acid–diol complexation. Tetrahedron.

[18] Cilliers J.J.L., Singleton V.L. (1991). Characterization of the products of nonenzymic autoxidative phenolic reactions in a caffeic acid model system. J. Agric. Food Chem..

[19] Arakawa R., Yamaguchi M., Hotta H., Osakai T., Kimoto T. (2004). Product analysis of caffeic acid oxidation by on-line electrochemistry/electrospray ionization mass spectrometry. J. Am. Soc. Mass Spectrom..

[20] Saiz-Poseu J., Mancebo-Aracil J., Nador F., Busqué F., Ruiz-Molina D. (2019). The Chemistry behind Catechol-Based Adhesion. Angew. Chem. Int. Ed. Engl..

[21] Müller M., Urban B., Reis B., Yu X., Grab A.L., Cavalcanti-Adam E.A., Kuckling D. (2018). Switchable Release of Bone Morphogenetic Protein from Thermoresponsive Poly(NIPAM-co-DMAEMA)/Cellulose Sulfate Particle Coatings. Polymers.

[22] Liebscher J., Mrówczyński R., Scheidt H.A., Filip C., Hădade N.D., Turcu R., Bende A., Beck S. (2013). Structure of Polydopamine: A Never-Ending Story?. Langmuir.

[23] Pretsch E., Clerc T., Seibl J., Simon W. (1989). Tables of Spectral Data for Structure Determination of Organic Compounds. Chemical Laboratory Practice.

[24] Hartig S.M., Greene R.R., Dikov M.M., Prokop A., Davidson J.M. (2007). Multifunctional Nanoparticulate Polyelectrolyte Complexes. Pharm. Res..

[25] Müller M. (2014). Sizing, Shaping and Pharmaceutical Applications of Polyelectrolyte Complex Nanoparticles. Adv. Polym. Sci..

[26] Takahashi R., Narayanan T., Sato T. (2017). Growth Kinetics of Polyelectrolyte Complexes Formed from Oppositely-Charged Homopolymers Studied by Time-Resolved Ultra-Small-Angle X-ray Scattering. J. Phys. Chem. Lett..

[27] Liu X., Haddou M., Grillo I., Mana Z., Chapel J.P., Schatz C. (2016). Early stage kinetics of polyelectrolyte complex coacervation monitored through stopped-flow light scattering. Soft Matter.

[28] Harrick N.J. (1979). Internal Reflection Spectroscopy.

[29] Senff H., Richtering W. (1999). Temperature sensitive microgel suspensions: Colloidal phase behavior and rheology of soft spheres. J. Chem. Phys..

