# Supplementary material for: Thermoresponsive Catechol Based-Polyelectrolyte Complex Coatings for Controlled Release of Bortezomib
Source: Int J Mol Sci. 2019 Dec 2;20(23):6081. doi: 10.3390/ijms20236081 (PMC6928965; doi:10.3390/ijms20236081)
Supplement: Supplementary file 1 [file ijms-20-06081-s001.pdf]

Supporting Information (SI)

"Thermoresponsive Catechol based Polyelectrolyte Complex Coatings for Controlled Release of Bortezomib"

Berthold Reis [1,2], David Vehlow [1,2], Tarik Rust [3], Dirk Kuckling [3] and Martin Müller [1,2]

[1]Leibniz-Institut für Polymerforschung Dresden e.V., Department Polyelectrolytes and Dispersions, Hohe Straße 6, 01069 Dresden

[2] Technische Universität Dresden, Department of Chemistry and Food Chemistry, 01062 Dresden, Germany

[3] Universität Paderborn, Department of Chemistry, Warburger Str. 100, 33106 Paderborn

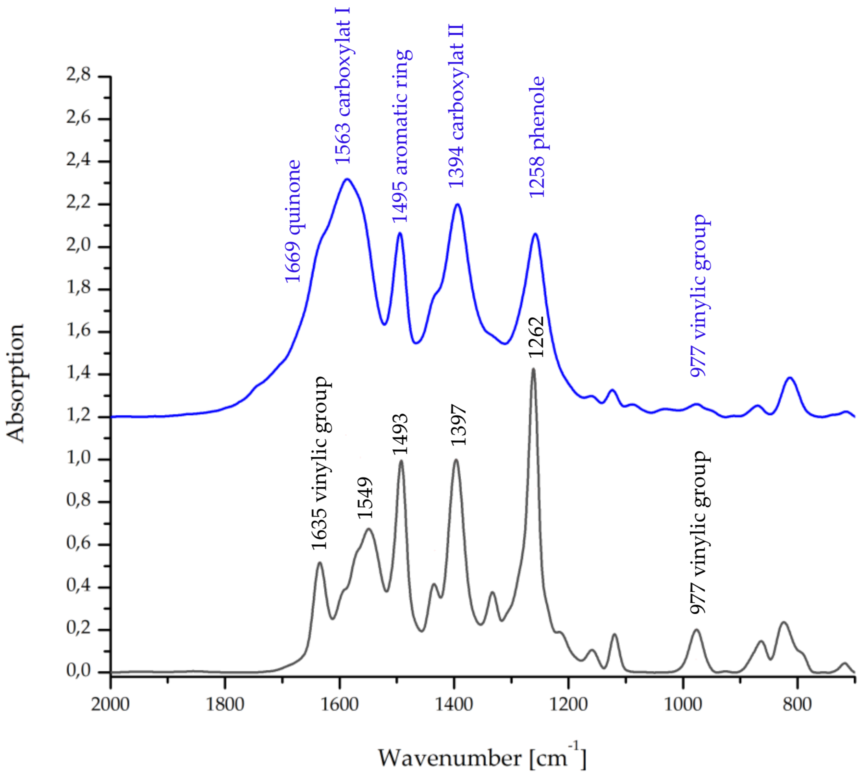

Figure S1. FTIR-spectra of the monomer CA (black) and the PCA (blue). Films were deposited onto Germanium substrates from 0,01 M solutions at pH = 9,0 and dried.

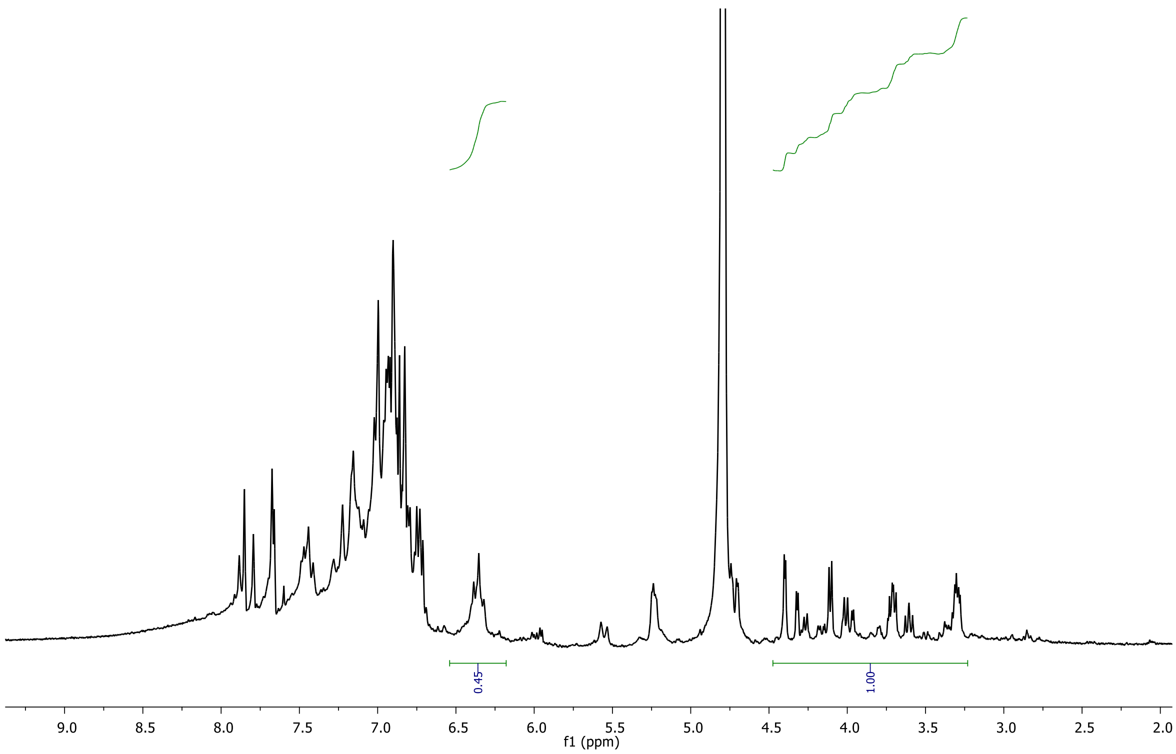

Figure S2. ^1H NMR (500 MHz, D_2O) spectra of PCA.

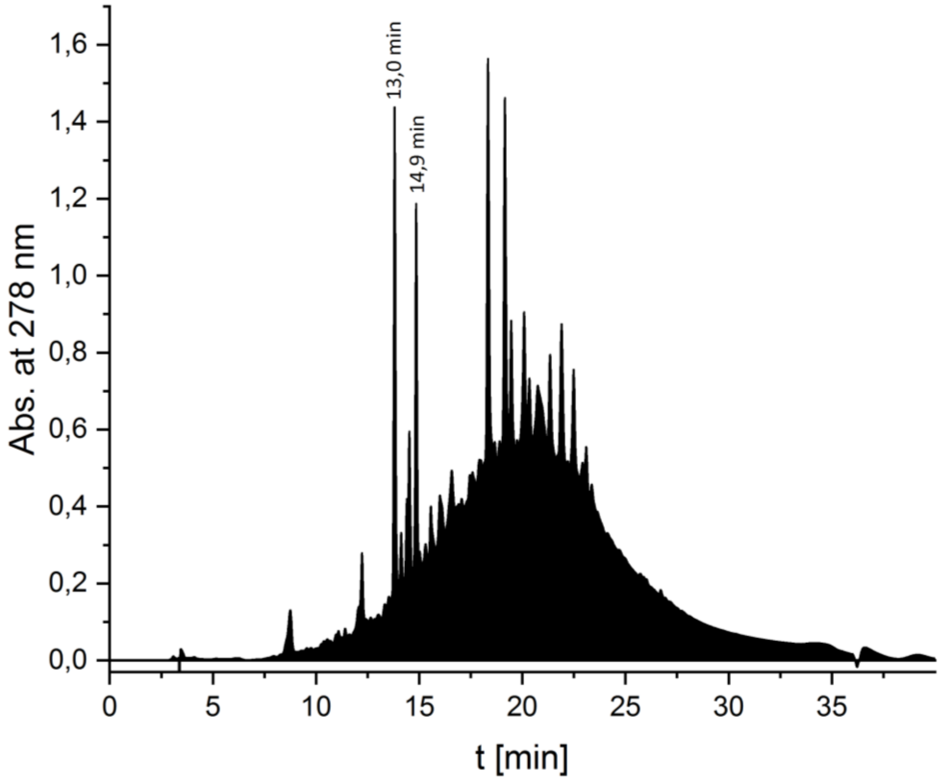

Figure S3. Chromatogram of reverse phase liquid chromatography of PCA, preferentially formed products at 13,0 min; 14,9 min; 18,4 min; 19,2 min and 22,0 min.

(**a**)

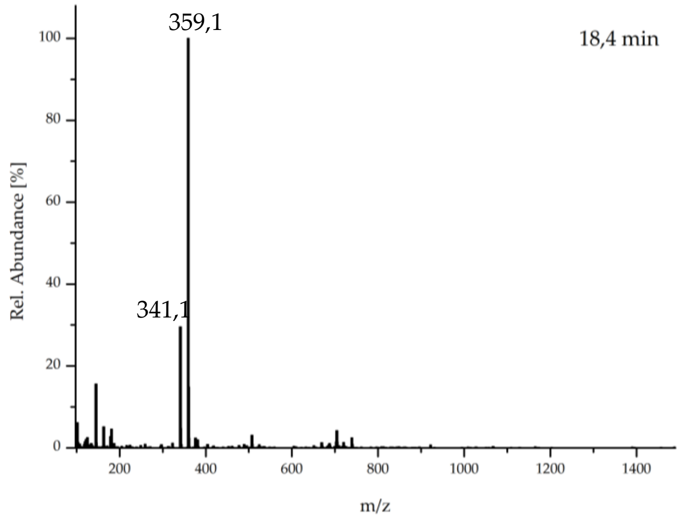

(**b**)

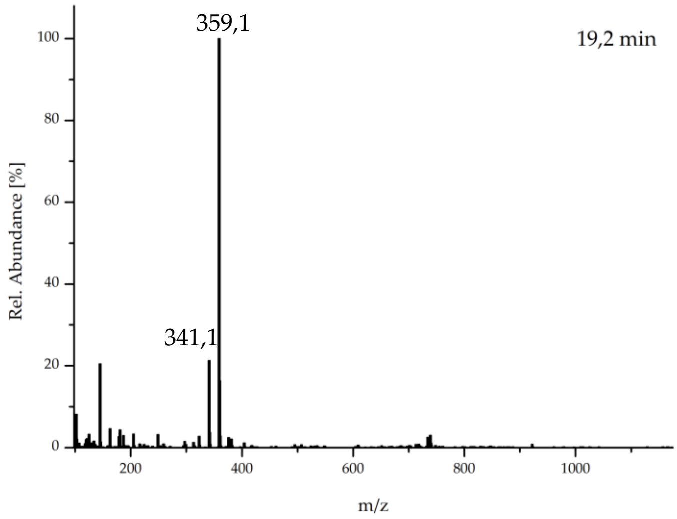

(**c**)

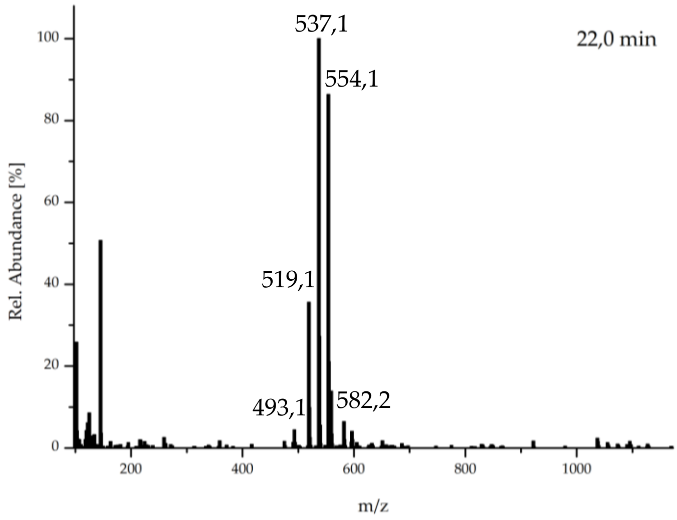

Figure S4. ESI mass spectra of the elution peaks (see Fig. SI3) at (a) 18,4; (b) 19,2 and (c) 22,0 min.

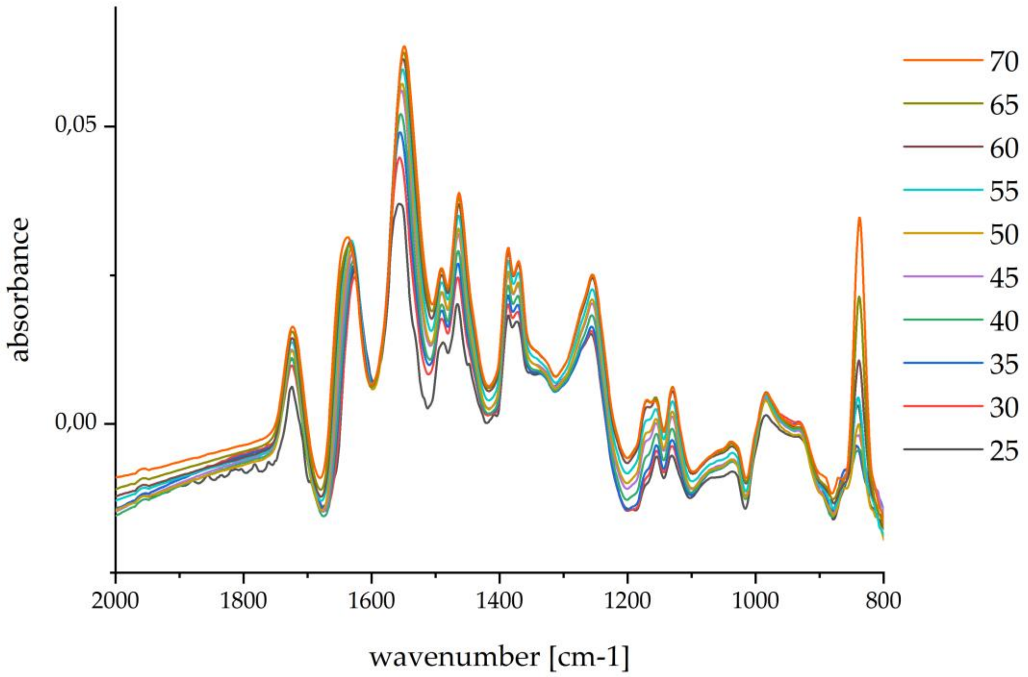

Figure S5. ATR-FTIR spectra of a P(NIPAM-*co*-DMAEMA)/PCA complex film at different temperatures. An increase is visible, especially of the amid II band (1560 cm^{-1}), from 25 °C (black) to 70 °C (orange).

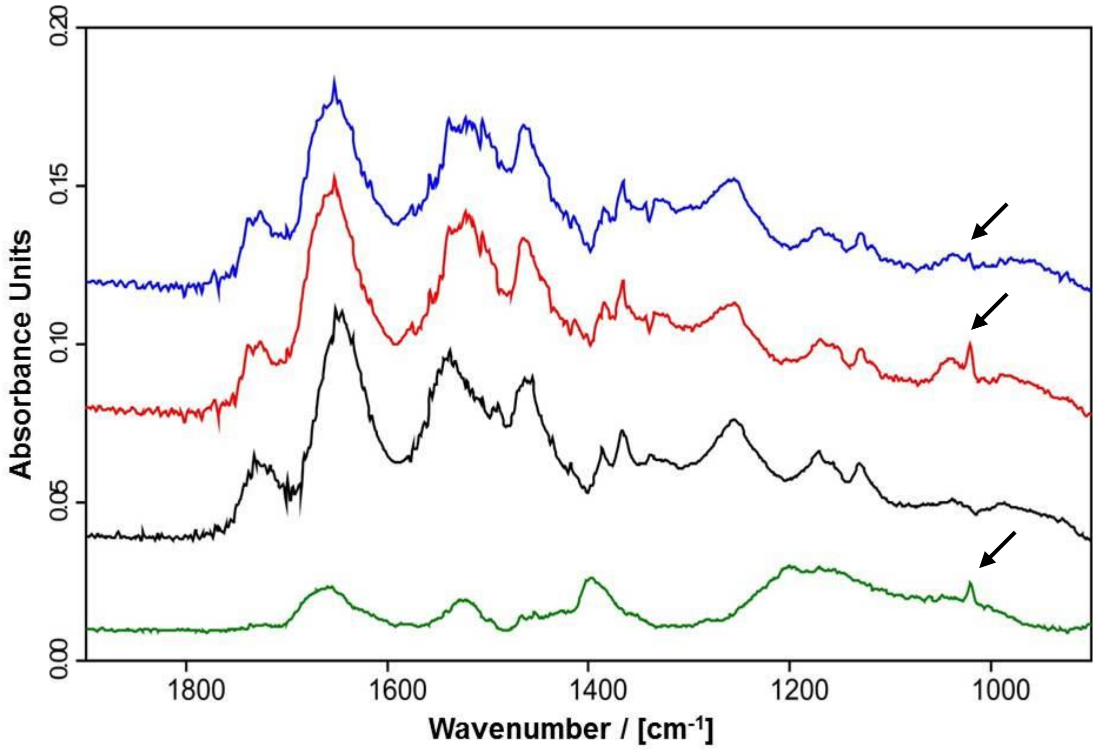

Figure S6. ATR-FTIR spectra of BZM film (100 µl from 0.25mM), P(NIPAM-*co*-DMAEMA)/PCA complex films before BZM loading (black), after BZM loading (red) and

rinsing in pure water (blue). Note the diagnostic band (arrow) of BZM (boron moiety) at around 1020 cm^{-1}.
